# Ethical Decision Making Levels of Nursing Students

**DOI:** 10.12669/pjms.343.14922

**Published:** 2018

**Authors:** Dilek Sari, Ebru Baysal, Gul Gunes Celik, Ismet Eser

**Affiliations:** 1 Dilek Sari, Associate Professor, Department of Fundamentals Nursing, Ege University School of Nursing, Izmir, Turkey; 2 Ebru Baysal, Research Assistant, Department of Fundamentals Nursing, Ege University School of Nursing, Izmir, Turkey; 3 Gul Gunes Celik, Research Assistant, Department of Fundamentals Nursing, Ege University School of Nursing, Izmir, Turkey; 4 Prof. Ismet Eser, Department of Fundamentals Nursing, Ege University School of Nursing, Izmir, Turkey

**Keywords:** Nursing, nursing students, Ethical decision making, Ethical dilemma, Ethics education

## Abstract

**Objective:**

The present study was conducted to determine nursing students’ levels of ethical decision-making.

**Methods:**

The sample of the descriptive study consisted of 240 nursing students. The data were collected using the Student Information Form and “Nursing Dilemma Test”.

**Results:**

It was found that Principled Thinking (PT) mean score (48.38±7.97) of nursing students was above average while their Practical Consideration (PC) mean score (17.87±4.13) was close to average. It was also determined that the nursing students participated in the study were not familiar (17.75±2.77) with the dilemmas included in the Nursing Ethical Dilemma Test.

**Conclusion:**

The students paid attention to consider ethical principles when making decisions about ethical dilemmas; however, they are also affected by environmental factors as well. Sex and class level were found to be influential in the process of ethical decision making.

## INTRODUCTION

Advances in science and technology have made patient care more and more complicated.^1^,^2^ As a result decision making responsibilities in nursing care to increase as well. Thus, nurses have begun to face many ethical problems such as initiating heart-lung resuscitation, ending a life-supporting treatment and patients rejecting treatment.^2^ Ethical decision making is a logical process which involves making the best moral decisions through systematic reasoning in a situation that brings about conflicting choices.^3^

Professional decisions of nurses affect their ethical problem solving skills and professional development of nursing students as well as the quality of patient care.^4^,^5^ If nurses fail to act in accordance with the principles taught at school in their practical applications, nursing students may fall into ethical conflict.^6^ Nursing students attend practical applications in the guidance of nurses and professors and make decisions in care. Therefore, nurses and professors must be role models for nursing students in clinical practices.^4^,^5^ Ethics education cannot be effective alone unless students have good role models. Practical norms and hierarchical structures may adversely affect new-graduates’ enthusiasm for ethical behavior.^7^

Studies conducted on nurses’ ethical sensitivity and ethical decision making levels have shown that they are not at the desired levels in ethical decision making.^8^,^9^ Likewise, studies carried out with student nurses have presented similar results.^3^,^10^,^11^ It has been found by studies on the ethical dilemmas experienced by nursing students and ethical decision making that the most frequent ethical dilemmas are telling the truth to incurable patients and their relatives,^4^ euthanasia, privacy,^10^,^11^ tapering of therapy,^4^,^11^ resuscitation of patients and patient rights.^11^

In order to improve nurses’ ethical decision making skills, they have to be undergo through basic education.^2^ Courses on ethics need be included within nursing curriculum. In-service training should cover ethical thinking skills and decision making process after graduation.^1^,^12^ It will enable them to identify when faced with an ethical problem, make the decisions in conflicts of ethical principles and develop solutions.^13^

It has been seen that the related literature includes few studies on the ethical decision making levels of nursing students.^5^,^11^,^12^,^14^,^15^ Considering the importance and necessity of ethical decision making, we believe that our study in which we measured nursing students’ levels of moral reasoning and ethical decision making will contribute to the literature.

## METHODS

The present descriptive cross-sectional study was consisted of the 637 students receiving education and training in the second and fourth years of the College of Nursing in the academic year 2016-2017. The formula [n=(N*x*^2^*x*p*x*q)/(d^2^x(N-1)*+*t^2^*x*p*x*q)] was used to calculate the size of the sample over the population.^16^,^17^ The calculation showed that the appropriate sample size would be minimum 240 students (105 students from second year and 135 students from fourth year). A stratified random sampling method was used in selecting the students to be taken in the sample according to class.^16^

### Data Collection Student Information Form

The form included four questions on students’ personal characteristics (class level, age, sex) and six questions about ethics and ethical dilemmas.

### Nursing Dilemma Test

Nursing Dilemma Test was developed by Patricia Crisham in 1981. The Turkish version of the test was analyzed for validation and reliability by Cerit (2010). The test formulated six scenarios. In each scenario, a situation is presented to possibly generate moral confusion for nurses offering care to the patient and family. The ethical dilemmas include: (a) newborn with anomalies considering the issue of defining and promoting the quality of life; (b) forcing medication; (c) adults’ requests to die; (d) orientation of a new nurse; (e) medication errors and (f) terminally ill adults. Each of the ethical dilemmas consists of three sections. The first section asks about the necessary action to be taken in case of the ethical dilemma given in the scenario and wants the answerer to mark one of the three options provided for each ethical dilemma. In the second section, six statements are presented which could be taken into consideration in the approach to the given scenario including the ethical dilemma. The participants are asked to choose the most important statement among these six and to put the statements in order of importance for themselves. The responses given in this section of the test aim at determining the levels of Principled Thinking” (PT) and “Practical Consideration” (PC). The possible minimum PT score on the test is 18, while the maximum PT score is 66. The lowest PC score that could be obtained on the test is 6 and the highest PC score is 36. PT shows the importance attached to considering moral principles when making a moral decision in nursing. PC, on the other hand, measures the importance given to environmental factors such as the number of patients, the number of available resources, institutional policies, the degree of nurses’ perception of the support given by the administration and the doctor’s control when making decisions about ethical problems. In the third section, the participants are asked to state whether they have any past experience with a similar dilemma or not. Based on the answers given to the question in this section, the state of having experience with a similar dilemma was assessed on a likert type scale and the “Familiarity” score was obtained. A familiarity score between 6 and 17 shows that the participants are familiar with a similar dilemma, while a score falling within the 18-30 range reveals no familiarity with a similar dilemma.^8^

### Ethical Considerations

Prior to data collection, an approval was obtained from X University College of Nursing Ethics Committee. Permission to use the Turkish version of the Nursing Dilemma Test was obtained from Birgül Cerit. Written approval was taken from school administrators to conduct the study. The study was conducted according to the Helsinki Declaration. Verbal consent was obtained from each student who agreed to participate after they were informed about the study content.

### Statistical Analysis

Statistical analysis was performed by using SPSS (version 17.0, SPSS Inc, Chicago, Illinois). General subject characteristics were analyzed using descriptive analysis through frequency, percentage, and means. Categorical variables were tested with Student t test and p<0.05 was considered statistically significant.

## RESULTS

It was found that the mean age of the students was 22.18 ± 1.41 years, a great majority of them (81.2%) were female and 18.8% were male. More than the half of the students (56.2%) was in their fourth year. Most of the students (62.9%) knew the definition of ethical dilemma, more than one third of them (38.4%) defined ethical dilemma as the conflict of values, very few students (6.2%) experienced ethical dilemma during their clinical internship and that the greatest ethical dilemma they experienced (33.5%) was in the case that the practices performed were different from what had theoretically been explained.

The data obtained from the second section of the Nursing Dilemma Test were evaluated and mean scores of PT, PC and familiarity that student nurses could get over this test were calculated. Accordingly, it was determined that the mean PT score 48.38±7.97 was a little above the average level, while the mean PC score 17.87±4.13 had a close value to average. The mean Familiarity score was 17.75±2.77 which showed that they were unfamiliar with similar dilemmas (Table-I).

**Table-I:** Mean Scores of Students in Nursing Dilemma Test.

Nursing Dilemma Test	M (SD)	Minimum	Maximum
Nursing Principled Thinking	48.38±7.97	27	62
Practical Consideration	17.87±4.13	8	30
Familiarity	17.75±2.77	6	30

The data obtained from section A of each scenario of Nursing Dilemma Test, 52.1% of the students were in favor of resuscitation of a newborn with abnormalities, nearly one third (31.2%) supported administering medication against the will of the patient while, and 38.8% of them remained undecided. As for the third scenario, 71.7% of the students stated that they would provide respiratory support although a competent adult patient requested to die. According to fewer than half of the students (40.4%) there is no time for the orientation of new nurses into the pediatric nursing clinic 40.4% remaining undecided. A great majority of the students (74.2%) stated that medication errors must be informed. The last scenario presented a dilemma about a terminally ill adult who asked his diagnosis despite his doctors’ and family members’ wishes. One third of the students (33.3%) thought that patients questions must be answered, 26.7% agreed with the doctor and family and 40.0% remained undecided (Table-II).

**Table-II:** Nursing student’s responses to section A of the Nursing Dilemma Test (n= 240).

Dilemmas	‘What should nurse do?’	n	%
Newborn with anomalies	Should resuscitate the newborn	125	52.1
	Cannot decide	73	30.4
	Should not resuscitate the newborn	42	17.5
Forcing medication	Should forcefully give the medication	75	31.2
	Cannot decide	93	38.8
	Should not forcefully give the medication	72	30.0
Adult’s request to die	Should provide assistance for artificial respiration	172	71.7
	Cannot decide	56	23.3
	Should not provide assistance for artificial respiration	12	5.0
New nurse orientation	Should allocate time for orientation of the nurse	97	40.4
	Cannot decide	97	40.4
	Should not allocate time for orientation of the nurse	46	19.2
Medication error	Should report the medication error now	178	74.2
	Cannot decide	48	20.0
	Should not report the medication error now	14	5.8
Terminally ill adults	Should answer the patient’s questions	80	33.3
	Cannot decide	96	40.0
	Should not answer the patient’s questions	64	26.7

While second year students’ Familiarity and PT scores were good, PC scores of the fourth year students were found to be higher (p<0.05). It was observed that female students’ Principled Thinking scores were high while male students had their PC scores higher (p<0.05). The students who knew the definition of ethical dilemma had higher Familiarity and PT scores whereas those who did not know the definition of ethical dilemma had higher PC scores (p<0.05) (Table-III).

**Table-III:** The Distribution of the Score of Nursing Dilemma Test by Several Variables.

		Principled Thinking Score			Practical Consideration Score			Familiarity Score		

Class	n	Mean±SD	T	P	Mean±SD	t	p	Mean±SD	t	p
Second year	105	51.09±6.86	4.955	0.000	16.92±3.75	-3.257	0.001	18.21±1.60	2.523	0.012
Fourth year	135	46.28±8.17			18.61±4.27			17.38±3.38		
*Sex*										
Male	45	45.31±8.37	-2.914	0.004	20.11±4.83	3.574	.001	17.57±3.48	-.384	0.702
Female	195	49.09±7.73			17.35±3.78			17.78±2.59		
*To know definition of ethical dilemma*										
Knowing	151	49.64±7.30	3.117	0.002	17.19±3.85	-3.408	0.001	18.04±2.13	2.171	0.031
Unknowing	89	46.24±8.63			19.03±4.34			17.24±3.57		

## DISCUSSION

When the ethical dilemmas experienced by nursing students in their clinical practices were examined, it was seen that the most frequently faced dilemmas included the fact that clinical practices did not comply with the standards in theoretical education, not telling the truth to the patient and making the decision of not applying CPR. These findings are similar to the results of previous studies carried out with student nurses.^4^,^11^

Principled Thinking show the importance attached to considering moral principles when making a moral decision in nursing. The present study found that mean PT scores of nursing students were a little above the average level (Table-I). In this respect, it could be said that - albeit not at the desired levels-student nurses think by taking ethical principles into consideration when faced with ethical problems. In some studies conducted with nurses,^9^,^12^,^15^,^19^,^20^ and nursing students^5^,^12^,^18^,^15^ PT scores were found to be above average, which supports our findings as well.

Moreover, students’ mean PT scores vary by their class levels, sex and their knowledge of the definition of ethical dilemma in the study. It was found that fourth year students, male students and those who fail to define ethical dilemma had lower mean PT scores (p<0,05) (Table-III). Fourth year nursing students are expected to have faced more ethical dilemmas and solutions since they have more experience in clinical practices.^12^ However, our study found that second year nursing students had higher mean PT scores than those students in their fourth year. As ethics courses are included in the first and second years in our school curriculum, this difference may have been affected by the fact that second year students have more recent knowledge of ethics. The study conducted by Ham (2004) support those obtained in our study. In the study they carried out with experienced nurses and graduate nursing students, it was found that the more years of experience the nurses had, the lower their mean PT scores got.^18^ On the other hand, Crisham (1981) stated that class levels of nursing students did not affect their man PT scores while Park et al. (2003) showed that PT scores of fourth year students were higher.^6^,^19^ In their study, Kurt et al. (2013) determined that male students had lower PT scores, but that their scores did not vary by class level.^5^

Practical consideration measures the importance given to environmental factors such as the number of patients, the number of available resources, institutional policies, the degree of nurses’ perception of the support given by the administration and the doctor’s control when making decisions about ethical problems.^8^ It was found in the present study that students’ mean PC scores were close to average (Table-I). Based on this finding, it could be asserted that students relatively remain under the influence of environmental factors when making decisions about the solutions of ethical dilemmas. Mean PC scores in our study are similar to those obtained in previous studies.^12^,^14^,^21^ On the other hand, in two different studies conducted with students^5^ and one study with nurses, mean PC scores are higher than those obtained in our study.^8^

In addition, mean PC scores of the students vary by their class level, sex and their knowledge of ethical dilemma. It was found that fourth year students, male students and those who were unable to define ethical dilemma had significantly higher mean PC scores (p< 0,05) (Table-III). Since they have more experience in clinical practice, fourth year students are considered to include environmental factors into the evaluation process when making decisions on ethical dilemmas.

It is seen in the study that, students with lower PT scores have higher PC scores. This could have resulted from the fact that the students are not familiar with similar ethical dilemmas. Familiarity may help nurses be able to discuss an ethical problem they are faced with, find appropriate solutions and make ethical decisions. Being unfamiliar with ethical dilemmas make is difficult to make ethical decisions and cause the nurse to use practical thinking.^5^ In our study, students’ mean familiarity score was 17.75±2.77, which showed that they were not familiar with similar ethical dilemmas (Table-I). In this respect, it could be said that students do not face with situations that are similar to those included in the scale in their real-life clinical practices. One study with students support our findings, while in studies conducted with nurses^4^,^9^ it is seen that nurses come across similar dilemmas more frequently in their clinical practices.

Also, familiarity scores of the students vary by their class level, sex and their knowledge of ethical dilemma. It was found that second year students, and those who were able to define ethical dilemma had significantly higher mean familiarity scores (Table-III).

It can be said that familiarity scores of the fourth year students might be affected by the fact that although they have more clinical experience, they took ethical courses in their second year.

When the responses of the students given to the sample ethical dilemmas in the scenarios were looked into. (Table-II), it was seen them most students assess patients’ well-being with a paternalist point of view and support survival instead of respecting patient autonomy and quality of life. In the field of healthcare, the concept of paternalism is mostly used to refer to professionals who restrict others’ autonomy in order to protect them from an anticipated or perceived damage. Sometimes professionals tend to make a dangerous assumption that they are the only ones who can make decisions on healthcare due to their professional knowledge and even that the sole knowledge required to decide for patients is professional knowledge.^22^ Previous studies showed that nursing students act as supporters of patient rights and behave more traditionally instead of using autonomy in the process of ethical decision making.^23^ Although students sometimes support the wholeness of the patients and their rights to designate their own destiny, they may perceive their authority insufficiently as students and fail to take action. Moreover, they may be afraid to be accused by others when they consider the patient’s will and personal values.^18^ In addition, nurses are officially responsible for fulfilling the written duties by doctors in Turkey. It is not the nurses’ legal obligation to inform the patient about the disease due to the respect for patient rights and, answering the questions about the diagnosis or situation of a terminally ill patient is beyond the official duties of a nurse in Turkey. Because of all these restrictions, nurses may think that they would be unable to make their own ethical decisions when they are faced with ethical dilemmas due to environmental conditions or professional and official responsibilities.^9^ These reasons may have caused the students to remain undecided about the given scenarios.

## CONCLUSION

The dilemmas most frequently faced by the student nurses included the fact that clinical practices did not comply with the standards in theoretical education, not telling the truth to the patient and making the decision of not applying CPR. Student nurses, albeit rarely, face with ethical dilemmas in the clinical environment, but that they are not familiar with similar ethical dilemmas from their previous experience. It was also concluded that they consider ethical principles at a level above average when deciding on ethical dilemmas and environmental factors are influential on their decisions. In our study, ethical decision making process of the students was found to be affected by sex, class level and the students’ knowledge of the definition of ethical dilemma. In order to improve nursing students’ critical thinking and ethical decision making skills, ethics courses must be included in the nursing curriculum and students need to discuss ethical dilemmas with their instructors during clinical practices. Ethics classes should be based on sample cases instead of explanation through traditional method to increase course efficiency and help students internalize them better.

### Authors’ Contribution

**DS, EB & GGC:** Designed the study, data collection and manuscript writing.

**DS & IE:** Designed the study, editing of manuscript, review and final approval of manuscript.
